# Elejalde syndrome - A neuroectodermal melanolysosomal disease: A case report

**DOI:** 10.22088/cjim.15.1.24

**Published:** 2024

**Authors:** Amir Hossein Noohi, Hossein Shojaaldini Ardakani, Kiavash Khashayar, Laily Najafi

**Affiliations:** 1 Department of Pediatric, Imam Ali Hospital, Alborz University of Medical Sciences, Karaj, Iran; 2 Endocrine Research Center, Institute of Endocrinology and Metabolism, Iran University of Medical Sciences, Tehran, Iran

**Keywords:** Elejalde syndrome, Neuroectodermal melanolysosomal disease, Silvery-gray hair, Silvery hair syndrome

## Abstract

**Background:**

Elejalde syndrome is a rare neuroectodermal melanolysosomal disease with an autosomal recessive heredity. Patients usually present with silvery-gray hair, neurological abormalities, diffuse skin hypopigmentation and suntanned skin color.

**Case Presentation:**

A 3 1/2-year-old boy presented with hemiplegia since the day before admission. Durig hospital admission, he experienced episodes of status epilepticus and loss of consciousness and underwent mechanical ventilation. The patient had silvery-gray hair, consequently the pathologic evaluation of the hair shaft, revealed enlarged irregularly spaced melanin clumps characteristic for silvery-gray hair syndrome. No immunologic dysfunction was detected due to immunological evaluations, subsequently Elejalde syndrome was confirmed.

**Conclusion:**

This study adds one new case to the known cases of Elejalde syndrome and confirms that Elejalde patients may not exhibit neurological symptoms until an older age.

Elejalde syndrome (ES) is a rare autosomal recessive disorder and known as neuroectodermal melanolysosomal disease (1, 2). Patients usually characterized by silvery-gray hair, central nervous system (CNS) dysfunction, and suntanned skin color. The CNS related problems of this disease include seizures, hypotonia, and mental retardation and are often severe (3). The defects can be either congenital or gradually progress during childhood. Silver colored hair and the suntanned skin color are prominently noticeable. In pathological analysis of the hair shaft in these patients, unevenly distributed large melanin granules are evident. Other pathological findings include abnormal melanocytes and melanosomes and inclusion bodies inside fibroblasts. The melanosome transport impairment is defined as the etiology of the skin hypopigmentation (3). However, they are less frequent findings. Autoimmune disorders do not occur in this disease (3-6). In the presenet report, a three and half year old boy of ES diagnosis is described.

## Case Presentation

A 3 ½ -year-old boy, presented with hemiplegia , ataxic gate and one episode of focal seizure as left sided gaze and some spasticity since the day before and was referred to Emam Ali Hospital (a tertiary care teaching hospital), Alborz, Iran. Seizure was controlled by phenytoin. Brain ct was performed due to focal seizure that was normal.The child was well nourished but mildly underdeveloped regarding three years and in lethargic mental status. No history of trauma, diarrhea, recent intramuscular injection or convulsions have been detected. Parents were not relatives and the patient had no history of admission. He had also one sibling who was normal. He was product of cesarian section with no problem at birth.

On physical examination, the vital signs were stable, he had silver colored hair, eyelid lashes and eyebrows that were otherwise completely normal in pattern and texture. The patient had diffuse skin hypopigmentation and a suntanned skin color in sunlight-exposed areas and had a light skin tone in other areas (figure 2A). The case had mildly under-developed mental status using Denver II test regarding his age, and recent right-sided hemiplegia; also, he had hypoactive deep tendon reflexes on his right side (upper and lower limb). Other sensory and motor neurological evaluations were normal. Ophthalmologic evaluation of the conjunctiva, cornea, lenses, retina, and optic disc in both eyes were normal, and the pupils were symmetrical and reactive to light bilaterlly. The patient had mild nystagmus.The patient had no history of admission due to previous infections, seizures, fever, trauma or any other problem till the present admission. The patient had one other sibling who was normal and his parents were nonconsanguineous. He was born at term by cesarian section with birth weight of 3250gms, and fully immunized.

Based on the examinations, the present patient was admitted with an impression of a probable cerebrovascular accident .While during hospital admission, he experienced episodes of status epilepticus and loss of consciousness, so, he was transferred to Pediatric Intensive Care Unit (PICU) and underwent mechanical ventilation. Before PICU admission, he was administered several drugs for focal seizures such as phenytoin, levetiracetam, diazepam, topomax and phenobarbital. All combinations failed to control seizures and led to focal status epilepticus. Unfortunately he developed a severe right sided hemiplegia after these episodes of refractory focal seizures not alleviated with time. After three days of pediatric intensive care unit hospitalization, the seizure episodes finally were controled, and the mechanical ventilation was halted. Due to mechanical ventilation, the patient developed ventilator-associated pneumonia (VAP) and was successfully treated with antibiotics.

**Figure 1:**
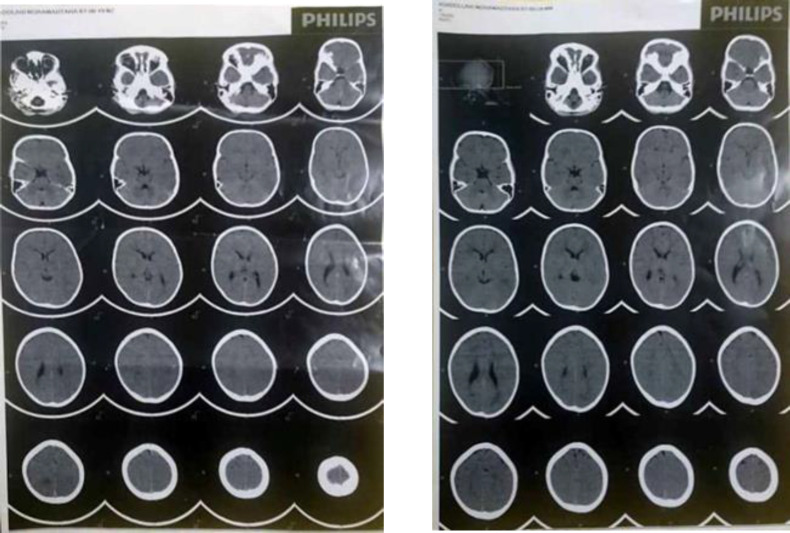
Brain MRI of three and half year old boy (signal change in external capsule, arrested hydrocephaly and hypomyelination in basal ganglia)

**Figure 2A, 2B. 2A:**
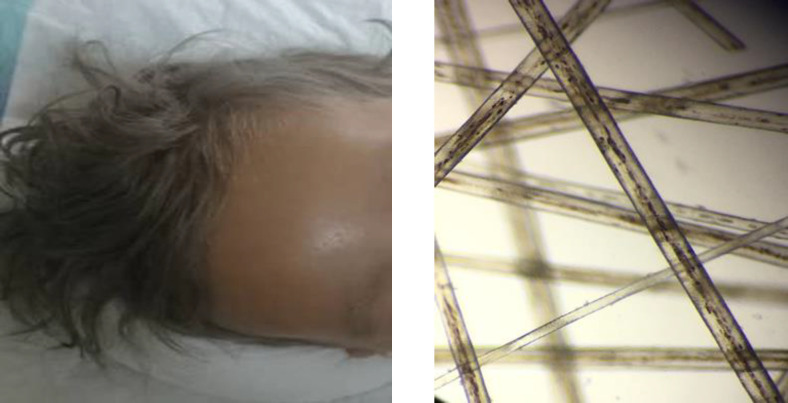
A three and half year old boy with silvery-gray hair, eyelid lashes and eyebrows. 2B: Pathological evaluation of the hair shaft by light microscopy, revealed enlarged irregulary spaced melanin clump (×400)

During his admission, laboratory evaluations were conducted. Serum hemoglobin level, hematocrit, platelet count, lipid profile, liver function tests, coagulation tests, electrolytes were all in normal ranges. The patient had leukocytosis but normal peripheral blood smear, and a rise in acute phase reactants such as c-reactive protein (CRP) that were related to VAP complication (Table 1). Brain MRI was performed that revealed signal change in external capsule, arrested hydrocephaly and hypomyelination in basal ganglia. Other parts were normal (figure 1). Light microscopy of the hair shaft revealed enlarged and unevenly spaced melanin clumps (figure 2B). With the assumption of the probability of Griscelli syndrome, the immunological consult was performed. Immunological evaluations were requested, and the findings revealed that the immunological function (humoral and cellular) is preserved (table 1). 

A comprehensive interview and the genetic consult did not reveal any similar cases in the pedigree. The patient was recommended to perform genetic evaluations, but his parents refused to sign the informed consent. Also his parents did not give permission for skin biopsy. The patient was discharged with a final diagnosis of ES, and anticonvulsant drugs (phenobarbital and levebel). His parents were informed and educated about the disease. In the later follow-ups, the patient passed away about 6 months after discharge when he was four years old due to frequent seizures, loss of consciousness and regressive neurologic process.

This case-report was accepted by the Ethics Committee of Alborz University of Medical Sciences; ethical code: IR.ABZUMS.REC.1400. 328. Detailed written informed consent was obtained from the patient’s parents. 

**Table 1:** Laboratory and immunological evaluations on the first hospital admission

**Laboratory findings**		**Values reference**
**CBC with diff**	WBC ((×1000/mm^3^)	8.72 5.5-15.5
	Neutrophils ((×1000/mm^3^)	6.25 (71.7%) 1.5-7.5
	RBC (millions/mm^3^)	5 3.8-5.2
	HGB (mg/dL)	12.7 10.5-14.5
	MCV (LfL)	71.6 74-94
	PLT (×1000/mm^3^)	241 150-470
**Electrolytes**	Na (mmol/L)	130 135-145
	K (mmol/L)	3.97 3.5-5
	Ca (mg/dL)	9.82 9.4-10.8
**Coagulation indices**	Homocysteine	6 3.4-11
	Thrombin (%)	90 80-125
	Anti-phospholipid IgG (IU/mL)	4.11 0-10
	Anti-phospholipid IgM (IU/mL)	2.16 0-12
	Protein C (IU/dL)	81 65-135
	Protein S (IU/dL)	57 60-150
	Total Anticardiolipin Antibody (IU/mL)	6.5 <12
	PT	15.1 11-13.5
	PTT	50 25-35
	INR	1.2 0.9-1.1
**Acute phase reactants**	CRP (mg/L)	41.2 0.02-14.4
	ESR (mm/h)	2 <10
**VBG**	pH	7.45 7.35-7.45
	pCO_2 _(mmHg)	32.5 35-45
	HCO_3 _(meq/L)	23.6 19-25
**Immunological indices**	F.A.N.A (titer)	0.17 <0.9
	NBT (%)	98 >90
	Diphtheria Antibody (IU/mL)	0.05 >0.1
	Tetanus Antibody (IU/mL)	0.04 >0.1
	Albumin (g/dL)	2.74 3.8-5.4
	C_3_ (mg/dL)	148 90-180
	C_4 _(mg/dL)	21.8 10-40
	Serum IgG (mg/dL)	633 635-1741
	Serum IgM (mg/dL)	192 45-281
	Serum IgA (mg/dL)	80.23 66-433
	CH50 (IU)	132 70-150

WBC: White blood cell; RBC: Red blood cell; HGB: Hemoglobin; PLT: Platelet; PT: Prothrombin time; PTT: Partial thromboplastin time; INR: International Normalized Ratio; CRP: C-reactive protein; ESR: Erythrocyte sedimentation rate; FANA: Fluorescent antinuclear antibody test; NBT: Nitroblue tetrazolium.

**Table 2:** Differentiation of syndromes with Silvery hair

**Elejalde**	**Chediak-Higashi**	**Griscelli**	
Normal	Phagocytic immunodeficiency	Phagocytic immunodeficiency	**Immunodeficiency**
Clumps of melanin in irregular patterns	Regularly arranged clumps of melanin	Clumps of melanin in irregular patterns	**Hair**
Melanosomes in different stages in melanocytes	Large melanosomes in melanocytes and keratinocytes	Mature melanosomes in melanocytes and sometimes in keratinocytes	**Skin**
Normal	Bleeding tendencies	Normal	**Bleeding**
Severe neurologic impairment (eg, seizures, severe hypotonia, mental retardation)	Progressive sensory or motor neurological defect	Failure of decussation of the optic and auditory nerves	**Neurological**

## Discussion

ES was first introduced in 1979 by Elejalde et al with the prevalence of <1/1000000. The etiology of ES is still unknown. Although several studies still neglect Elejalde et al.’s discovery and classify silvery-gray hair, immunopotent patients with neurological problems as a subclass of Chediak-Higashi or Griscelli syndromes type I (6-8).

Silvery-gray hair and pigmentary abnormalities are manifestations of a group of extremely rare autosomal recessive genetic disorders. This group consists of three main diseases; Griscelli syndrome, Chediak-Higashi syndrome, and ES. Griscelli syndrome is characterized by hypopigmentation with immunodeficiency that usually causes death by early childhood. In comparison, Chediak-Higashi patients tend to bruise and bleed easily in addition to Griscelli syndrome's manifestations. ES, is the rarest syndrome of this group with very few cases reported so far. Unlike Griscelli and Chediak-Higashi, ES does not cause immune impairment (table 2). Even though inclusion bodies are seen in fibroblasts, lymphocytes, and bone marrow in ES, they differ from the inclusion bodies seen in Chediak-Higashi syndrome. Presumably, the melanin in the skin and hair and neuromelanin present in the brain plays a crucial role in the pathological changes of ES. Neuromelanin is a key molecule attributed recently in Parkinson and other neurodegenerative disease. The role of this complex molecule is unknpwn and we don’t know wether structure or function is involved in pathogenesis of E.S. Probably neurodegeneration due to unequal distribution of melanin in CNS may be the main etiologic factor (5).

In McKinster et al.’s study, seven ES cases were presented, in which; 4 out of 7 of them were significantly retarded since birth and had congenital pattern, the other three patients suffered from a regressive neurological process after birth and their first signs developed during childhood. McKinster et al. demonstrated the first research showing that children suffering from ES could develop cognitive problems after several years of living (5). The present study adds another case to ES patients with noncongenital neurological problems.

The pathological study of the hair shaft is the critical diagnostic factor of ES. The melanin in ES patients' hair shafts is unevenly distributed. This uneven distribution and having areas without any melanin results in the bright silver-colored hair. Pathological studies can be useful in differentiating the diseases that have silvery-gray hair manifestation (9).

The characteristic diagnostic criteria of Chediak-Higashi syndrome in the hair analysis is regular small melanin granules distributed in both cortical and medullar zones. In contrast, the characteristic pattern of Griscelli and Elejalde syndromes, are consisting of irregularly distribution of small and large melanin clumps along the hair shaft (9-11).

ES cases in McKinster et al.’s study, suffered from nystagmus, diplopia, and congenital amaurosis in addition to the skin and common neurological manifestations of this disease (5). The present case did not exhibit any ophthalmological symptoms, which were also confirmed by the ophthalmological examination. The probability of missing the cases until an older age should be considered such as the present case and Ivanovic study, who reported a 12 years old male with ES (2).

Approximately half of the reported cases expired in early childhood (2, 5, 6, 12). A genetic counseling for future pregnancies is an appropriate tool that may be offered to families who already suffered Elejalde children, considering lack of proper treatment for these patients. Further studies on the pathogenesis and the etiologies of this disease may open new windows in the treatment and management of these patients (9).

The current study adds one new case to the known cases of ES and confirms that Elejalde patients may not exhibit neurological symptoms until an older age. Earlier detection and diagnosis of the disease may lead to better patient management and prevent an affected sibling birth.
